# Selective observation of the disordered import signal of a globular protein by in-cell NMR: The example of frataxins

**DOI:** 10.1002/pro.2679

**Published:** 2015-04-10

**Authors:** Matija Popovic, Domenico Sanfelice, Chiara Pastore, Filippo Prischi, Piero Andrea Temussi, Annalisa Pastore

**Affiliations:** 1 National Institute for Medical Research, MRC, The RidgewayLondon, United Kingdom; 2 Department of Clinical Neuroscience, King's College London, Denmark Hill CampusLondon, United Kingdom; 3 Department of Life Sciences, Centre for Structural Biology, Sir Ernst Chain Building, Imperial College LondonLondon, SW7 2AZ, United Kingdom

**Keywords:** flexibility, frataxins, in cell, intrinsically unfolded proteins, NMR

## Abstract

We have exploited the capability of in-cell NMR to selectively observe flexible regions within folded proteins to carry out a comparative study of two members of the highly conserved frataxin family which are found both in prokaryotes and in eukaryotes. They all contain a globular domain which shares more than 50% identity, which in eukaryotes is preceded by an N-terminal tail containing the mitochondrial import signal. We demonstrate that the NMR spectrum of the bacterial ortholog CyaY cannot be observed in the homologous *E. coli* system, although it becomes fully observable as soon as the cells are lysed. This behavior has been observed for several other compact globular proteins as seems to be the rule rather than the exception. The NMR spectrum of the yeast ortholog Yfh1 contains instead visible signals from the protein. We demonstrate that they correspond to the flexible N-terminal tail indicating that this is flexible and unfolded. This flexibility of the N-terminus agrees with previous studies of human frataxin, despite the extensive sequence diversity of this region in the two proteins. Interestingly, the residues that we observe in in-cell experiments are not visible in the crystal structure of a Yfh1 mutant designed to destabilize the first helix. More importantly, our results show that, in cell, the protein is predominantly present not as an aggregate but as a monomeric species.

## Introduction

Direct observation of folded proteins inside cells by nuclear magnetic resonance (NMR) techniques, an approach originally introduced by Doetsch *et al*.,^1^ provides an attractive potentiality that could allow the observation of proteins in their natural *milieu* without lengthy and intrusive steps of purification. It was, however, recently realized that the spectra of globular proteins are often not observable, probably because of non-specific interactions and/or confinement^2^–^10^ which make molecular tumbling too slow. On the contrary, the spectra of intrinsically unstructured proteins are mostly observable when over-expressed in bacteria.^3^ This different behavior offers a unique possibility to distinguish between flexible parts and more rigid parts also when studying in-cell NMR of folded proteins.

Here, we applied this concept to compare the spectra and the flexibility in cell of two orthologues of the same protein family. Frataxins are small proteins (10−12 kDa) highly conserved in most organisms from bacteria to humans.^11^,^12^ They are essential components of the cell, taking part in iron sulphur cluster biogenesis through interactions with the Nfs1/Isu complex (or IscS/IscU in prokaryotes) that is central to this machinery.^13^ In prokaryotes, the structure of frataxin (called CyaY in bacteria) consists of a compact globular domain (100−120 amino acid in length) with a αβ fold, which is common to all species (pdb code 1ew4).^14^–^16^ In eukaryotes, frataxins are produced in the cytoplasm and successively imported in the mitochondria.^17^,^18^ The conserved globular domain is thus preceded by an N-terminal tail that is specific to eukaryotes and is responsible for mitochondrial import (pdb code 2fql).^19^,^20^ A detailed study has suggested that *in vitro* the import signal of human frataxin overexpressed in *E. coli* is, as most mitochondrial import signals, intrinsically unfolded.^12^ However, evidence from the yeast ortholog (Yfh1) suggests a role for the import signal in the formation of spheroidal assemblies that have been suggested to ligate iron and keep it in a functionally available form.^21^,^22^ A crystal structure of a Yfh1 mutant in which Y73 was mutated to A73, supports this hypothesis in that it shows a trimer, that is the minimal component of the assembly.^23^ The trimer protomers pack against each other in such a way that their N-terminal region plays a crucial role in the stabilization of the trimer. The residual small helix located on the N-terminal part after the Y73A mutation has made the whole N-terminal more flexible, packs against the β sheet of the adjacent protomer. Formation of these assemblies seems to be modulated by the presence of oxygen as in strictly anaerobic conditions no assembly is observed.

To understand better the dynamical properties of frataxins we resorted to in cell NMR in the hope of observing frataxins from different species directly in cell. Here, we report a comparative study that helps to clarify the role of the N-terminal part and provide an example of the power of this technique when correctly tailored to the scientific question.

## Materials and Methods

### Sample preparation and optimization of the in-cell protocol

The two frataxin orthologues, cloned in pET21a vectors, were transformed in BL21(DE3) *E. coli* cells and selected for transformation in ampicillin plates, according to previously published protocols.^16^,^24^ CyaY comprised the full-length 106 amino acid protein sequence, whereas the Yfh1 construct contained residues 52−174 which corresponds to the mature form of Yfh1.^19^ Val52 was mutated to a methionine for molecular biology purposes. Single colonies were grown overnight at 37°C in 3 mL of Luria Broth medium and 2 mL of the overnight cultures were used to inoculate 100 mL of fresh medium. Different values of optical density between 0.6 and 1.0 were tried before deciding that a value of 0.8 was the best for all preparations. When the cells reached the desired optical density, they were harvested by centrifugation at 3580 g for 15 min at room temperature. The pellet was then resuspended in 100 mL of ^15^N enriched minimal medium, incubated in a rotary shaker at 37°C for 10 min and induced with IPTG (0.5 m*M*). These conditions were the result of exploratory work using IPTG concentrations in the range 0.02−1 m*M* and adaptation times in the range 10−30 min. The cells were harvested by centrifugation at 5000 rpm for 15 min after induction times varying from 1 h to 4 h. It can be appreciated (Fig. S1 Supporting Information) that there is a substantial gain in doubling the induction time from 1 h to 2 h. Longer times afforded no appreciable advantage. Shorter induction times proved systematically better. Following induction, the cells were washed once with 25 mL of the M9 solution, centrifuging for 15 min at 3850 g at room temperature. The final pellet was resuspended in 500 µL of M9 medium mixed with 50 µL of D_2_O, before transferring it into standard 5 mm NMR tubes using a Pasteur pipette and used immediately for NMR experiments.

To exclude the possibility that the NMR spectra stem from leakage to the supernatant, for each experiment we run spectra of the supernatant of the in-cell NMR samples and checked the actual content of harvested cells by lysing them by sonication. The in-cell NMR samples were spun at 10,000*g* for 5 min at room temperature and the supernatant used for the leakage control measurement. The pellet was resuspended as before in 500 µL of M9 medium mixed with 50 µL D_2_O and sonicated on ice for 3 min using a Branson sonifier 250 (duty cycle 40%, output power at level 5). The lysate was further centrifuged at 15,000*g* for 20 min at room temperature and its supernatant placed in the NMR tube. When specified, strict anaerobic conditions were obtained by lysing the cells in an anaerobic chamber (Belle Technology) under nitrogen and transferring the lysate to an NMR tube (5 mm internal diameter) closed with a rubber septum. In this case, cells were lysed by 10 freeze-thawing cycles on dry-ice. The pellet was resuspended in the anaerobic chamber using degassed lysis buffer (450 µL of M9 medium mixed with 50 µL D_2_O) supplemented with lysozyme and DNAseI (Roche) to reach the final concentrations of 1 mg/mL and 1 µg/mL, respectively.

### NMR experiments

HSQC and SOFAST experiments were typically recorded at 25−27°C and 600 MHz making sure that data collection would not exceed 10−15 min (but typically time was much shorter) to avoid cell lysis. Data processing was performed in Biopack (with linear prediction in the ^15^N dimension) and NMRPipe.^25^ The spectra were analyzed using CCPN software.^26^ To avoid leakage, careful sample handling was required to prepare the cell slurry.^27^

A standard NOESY experiment was carried out on purified Yfh1 using a mixing time of 100 ms. ^15^N T_1_, ^15^N T_2_ and heteronuclear ^15^N-[^1^H] NOE data at 25°C and 600 MHz on the Yfh1 lysate were measured at 600 MHz using standard pulse sequences. Overlapping resonances were not included in the analysis. An estimate of the overall rotational correlation time *τ*_c_ was obtained from the trimmed average *T*_1_/*T*_2_ ratio, i.e., excluding residues with *T*_1_/*T*_2_ values greater than one standard deviation from the mean.^28^

## Results

### CyaY is undetectable by in-cell NMR

**Figure 1:**
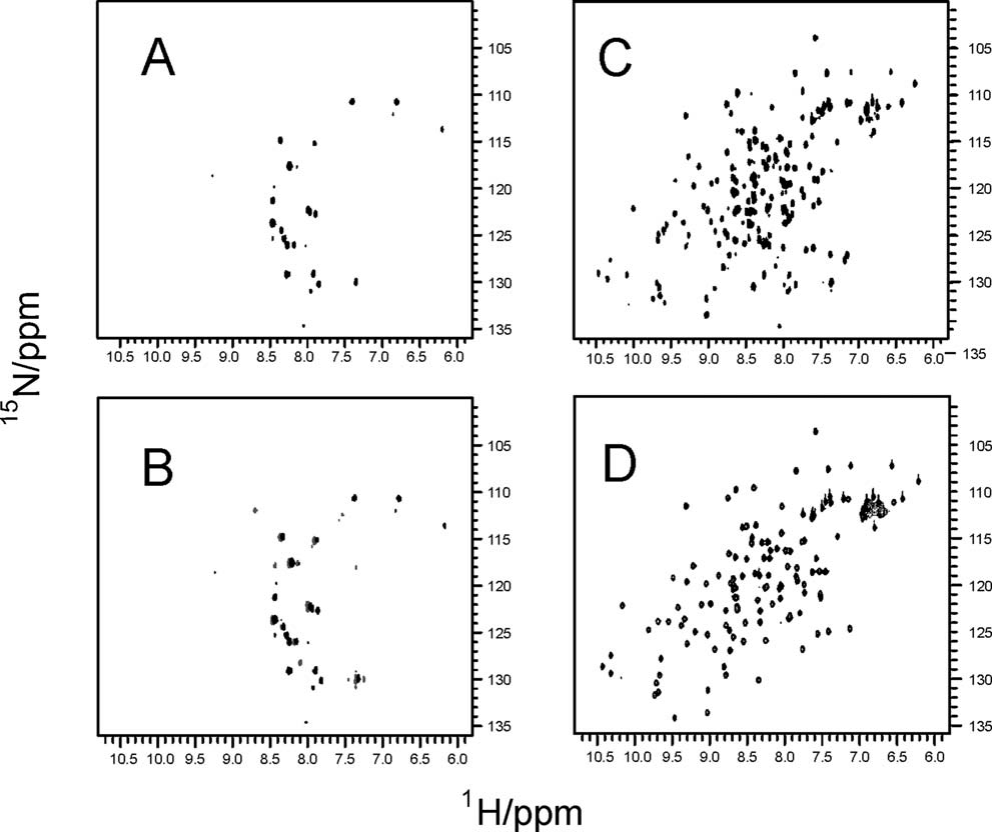
^15^N HSQC NMR spectra of CyaY in cell and in the lysate. A: Spectrum of whole cells suspension after induction of the protein. B: Superposition of spectra of whole cells suspension with and without protein induction. C: Spectrum of lysed cells after induction of the protein. D: Spectrum of the purified protein. All spectra were recorded at 600 MHz and 25°C. Comparable counter plot levels were used for all spectra.

When we overexpressed CyaY, the bacterial ortholog of frataxin, in *E. coli* the in-cell spectrum resulted not visible: the spectrum [Fig. 1(A)] contains very few peaks and these resonances are present also in the spectrum of the noninduced sample [Fig. 1(B)]. The spectrum is certainly that of a protein in cell because controls carried out by spinning down the cells and recording the spectrum of the supernatant did not show any protein signal (data not shown). The three most intense peaks have been recognized to belong to a metabolite and must thus be considered background.^29^ Conversely, when the induced cells were lysed by sonication, the spectrum reappeared in full [Fig. 1(C)], with features that are similar to the spectrum of the purified protein [Fig. 1(D)]. This behavior has been ascribed to weak interactions with other cellular components or to the effect of confinement that restricts the protein tumbling inside the cell.^2^–^9^ A difference between the two spectra is the presence of more peaks in the in-cell spectrum probably due to some degradation which we could reduce using blander lysis conditions but not eliminate. There are also noticeable variations of chemical shifts which are likely due to the different composition of the medium which is expected to have a strong influence particularly for members of the frataxin family since these proteins are able to bind several cations.^16^,^30^ Finally, the in-cell spectrum is overall more broadened as compared to the spectrum of the purified protein as expected for a protein in a more viscous medium.

### The N-terminal tail of yeast Yfh1 is flexible

**Figure 2:**
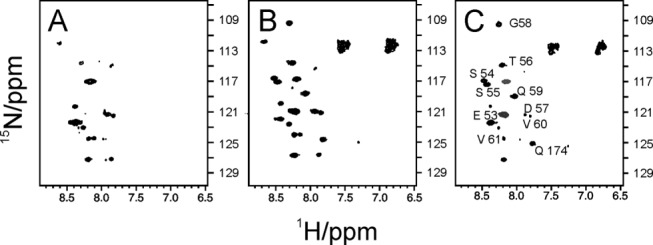
Comparison of the ^15^N HSQC NMR spectra of cells expressing Yfh1. A: Spectrum of whole cells suspension without protein induction. B: Spectrum of whole cells suspension after induction of the protein. C: Difference spectrum. Visible protein peaks originating from the N-terminal are shown in black. Gray peaks correspond to two very intense background resonances of panel A. The spectra were recorded at 600 MHz and 25°C.

**Figure 3:**
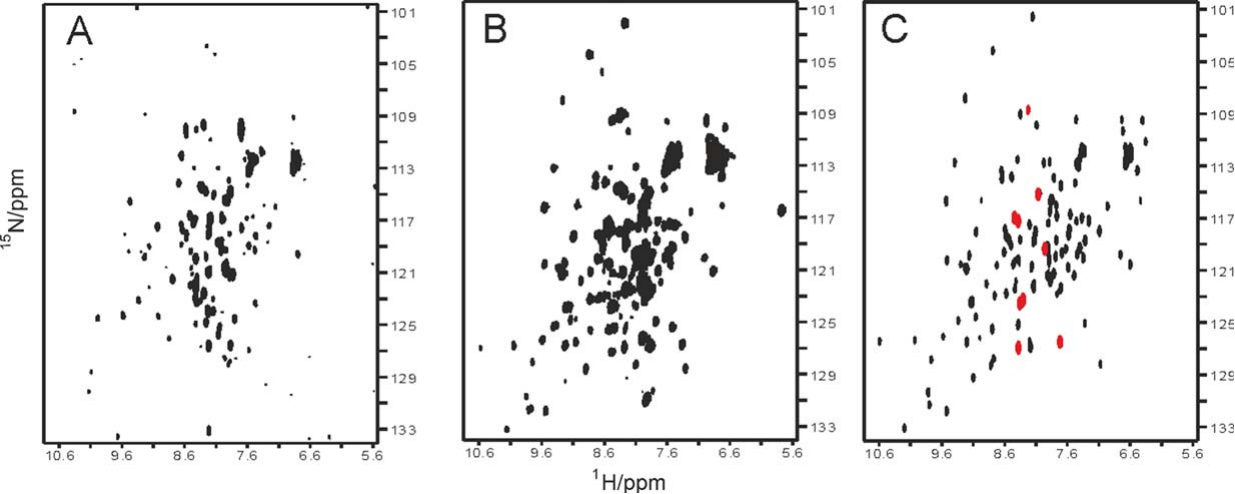
Comparison of the ^15^N HSQC NMR spectra of cells expressing Yfh1. A: NMR spectrum of Yfh1 cell lysate (1min sonication). B: NMR spectrum of Yfh1 cell lysate of harvested cells after spinning down membranes and other cell components. C: NMR spectrum of Yfh1 after purification. N-terminal peaks coincident with those of Figure 2(C) are colored in red.

We observed a similar scenario in the spectrum of Yfh1 but, in addition to the peaks of the metabolite, there are about nine clearly identifiable resonances that are absent in the noninduced control and that have thus to arise from the protein [Fig. 2(A,B)]. A control carried out by spinning down the cells and recording the spectrum of the supernatant did not show any protein signal (Fig. S2 Supporting Information). The difference spectrum shows that it is possible to eliminate nonrelevant background signals to isolate the peaks belonging to the N-terminus [Fig. 2(C)]. The remaining peaks can be assigned to residues from E53 to V61 of Yfh1 which belong to the unstructured N-terminal tail that is the remaining part of the mitochondrial import signal. Selective observation of the Yfh1 spectrum is in full agreement with the recent suggestion that in-cell NMR is easier for intrinsically unstructured chains that, being flexible, remain visible also in the *milieu* of the *E. coli* cytosol^3^. Interestingly, these residues could not be observed in the crystal structure of a mutant because they are part of the trimer interface.^23^ Also in this case, the spectrum was fully recovered after cell lysis [Fig. 3(A)], showing some broadening which becomes less pronounced after spinning down membranes and other cell components [Fig. 3(B)]. The spectrum is similar to that of the purified protein [Fig. 3(C)] except for some shifts and some additional resonances presumably due to the process of cell lysis. Peaks coincident with those of Figure 2(C) (from E53 to V61) are colored in red.

These results tell us that the N-terminus of Yfh1 is highly flexible and has dynamics different from the attached globular domain.

### Ordering starts around V61 of Yfh1

**Figure 4:**
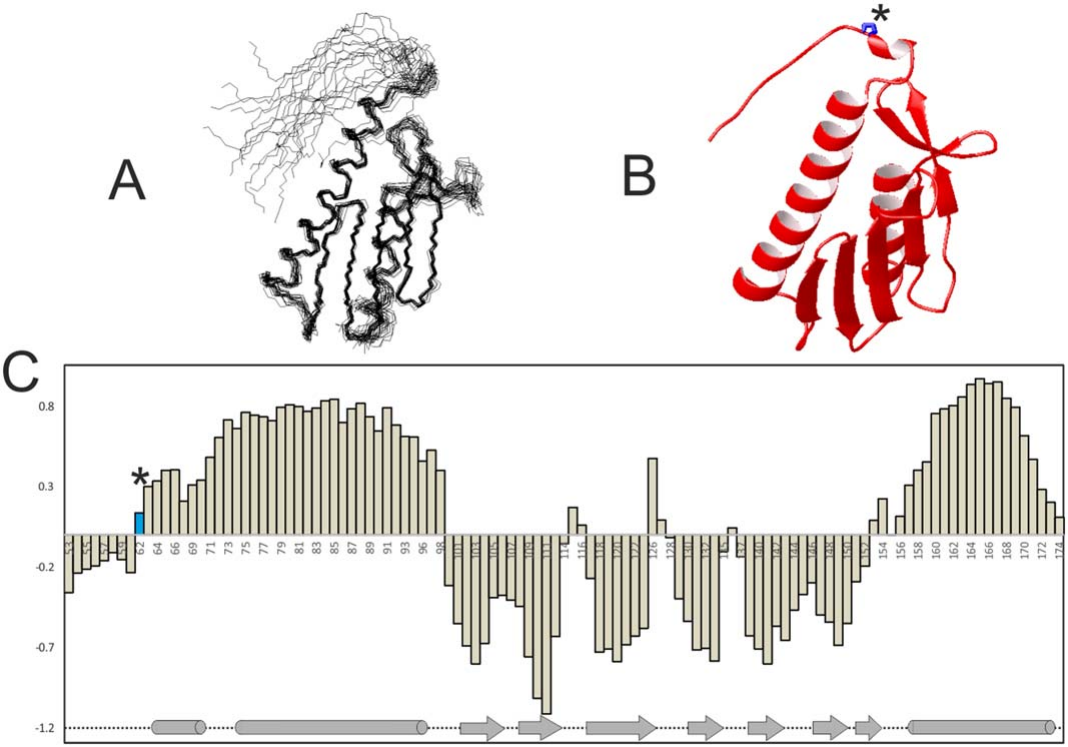
NMR structure and chemical shift analysis of Yfh1. A: NMR bundle of 20 structures (pdb id 2ga5). B: Mean structure of the 20 structures of Figure 4(B), represented as ribbon. The side chain of residue P62 is marked by an asterisk. Molecular models were generated by MOLMOL.^40^ C: Chemical shift indexes of Yfh1. The N-terminal region values are close to zero, as expected for a disordered segment. The bar corresponding to P62 is indicated in black. The secondary structure is indicated below using cylinders for helices and arrows for sheets.

To assess further the state of fold of the N-terminus, we acted on the lysate in which the spectrum of Yfh1 is visible and plotted the chemical shift indexes [Fig. 4(A)]. Using the assignment recently obtained,^24^ the first 10 residues have low chemical shift indexes although not completely null. The chemical shifts of the corresponding in-cell resonances are indistinguishable from these values indicating that the residues must have the same secondary structure in cell and in the lysate (Fig. 3).

We also analyzed a 3D ^15^N NOESY to test the presence of NOEs between the N-terminus and the globular domain according to what would be expected from an NMR structure (2ga5). We found no NOEs in the N-terminus other than those due to the primary structure up to residue N63. The amide of N63 forms a sequential NOE with the following amide (E64) suggesting the presence of a bending or a local turn [Fig. S3 and 4(B)]. These observations suggest the presence of local structure at this residue in an otherwise unstructured region.

### In cell, Yfh1 is predominantly a low molecular weight species

**Figure 5:**
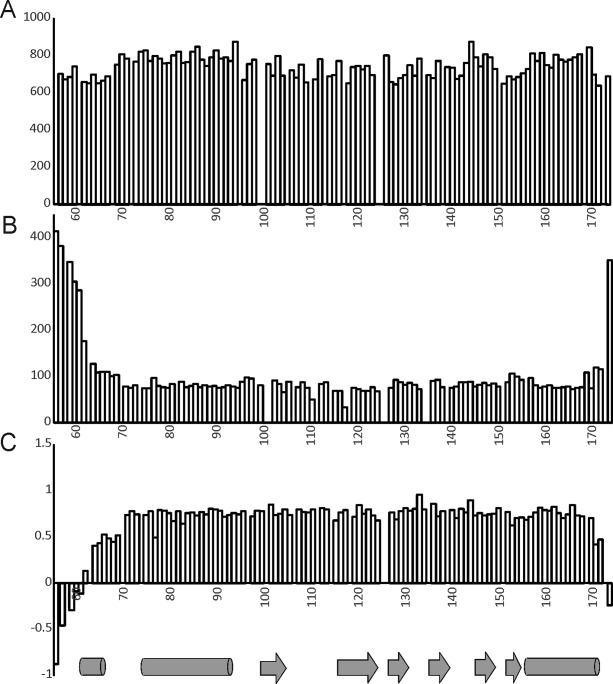
NMR relaxation parameters of Yfh1 in cell suspension as measured at 600 MHz and 25°C on a uniformly ^15^N-labeled sample of Yfh1 at 0.3 m*M* concentration. (A) *T*_1_ values (s), (B) *T*_2_ values (s), and (C) ^15^N-[1H] heteronuclear NOE. The secondary structure is indicated below using cylinders for helices and arrows for sheets.

Yfh1 has been suggested to form iron-promoted aggregates,^21^ raising the possibility that the absence of an in-cell spectrum could be explained by self-aggregation. We approached this hypothesis in different ways. We measured the relaxation rates to get a more quantitative idea about the difference of tumbling time of the protein when purified and in the lysate (Fig. 5). NMR relaxation measurements (*T*_1_, *T*_2,_ and hetero-nuclear ^15^N-[^1^H] NOE values) were recorded at 25°C and 600 MHz on a ^15^N uniformly labeled sample of Yfh1 to provide a measure of the local degree of flexibility. We observed a correlation time of 16.3 ms. This value corresponds approximately to a protein of about 24 kDa that is almost the value expected for a isotropically tumbling dimer of Yfh1 (the monomer is 13 kDa).^31^ For comparison, the correlation time calculated for CyaY in the lysate is 11.5 ms which corresponds to a protein of about 17.6 kDa as compared with the expected value of 11 kDa. Increased values in cell are expected because of the crowded environment. Additionally, while CyaY is globular and roughly isotropic, Yfh1 contains an unfolded tail which greatly increases the anisotropy of the protein. It was previously demonstrated that an unstructured tag of 11 residues increases of about 30% the correlation time of the titin domain I27.^32^ Our results thus exclude the formation of appreciable quantities of large aggregates since the observed values would barely account for a dimer. They could reflect a mixture between low and high molecular weight species in which, however, the formers would be largely predominant.

Finally, we reasoned that, because the aggregates of Yfh1 are known to be destabilized by aerobic conditions,^33^,^34^ they could be artificially disrupted in our assays. We thus measured the dynamical properties of the lysate breaking the cells under strict anaerobic conditions. The appearance of the spectrum and the line widths were indistinguishable from those obtained under aerobic conditions (data not shown). These results bring no support to the concept of ferritin-like aggregates at least in bacterial cells.

## Discussion

The initial impression from the first results of in-cell NMR^1^ was that it could be the ultimate solution to many of the problems of structural biology. In principle, it would allow structure determination of proteins in solution directly in cell, bypassing lengthy, invasive and possibly troublesome purification steps and, even more important, allowing the study of biological macromolecules directly in their cellular environment. The first, and so far only, protein structure determined inside a cell was that of TTHA1718, a signal protein from a thermophile organism.^35^ A few investigations did find differences, albeit small, between *in vitro* and in-cell NMR parameters. Serber *et al*.^1^ observed that the chemical shifts and relaxation rates of residues in the metal-binding loop of NmerA are slightly different from the corresponding ones of the *in vitro* state, suggesting possible biologically relevant variations in local conformation and dynamics. Hubbard *et al*.^36^ showed that in the case of CheY, a signaling protein, the mode of binding of a small molecular-weight compound was similar *in vivo* and *in vitro*. Burz *et al*.^37^ proposed a general use of in-cell NMR to map structural changes that accompany protein−protein interactions (STINT-NMR). Unfortunately, these aspects could be confirmed only in a few *ad hoc* cases. It has soon become clear that the behavior of the protein at the center of the study of Serber *et al*.,^1^ namely NmerA, was an exception rather than the rule. Most folded proteins, when overexpressed in bacteria, do not show an NMR spectrum. However, it was also noticed that intrinsically disordered proteins are invariably observable when overexpressed in bacteria. In a comparative study, Li *et al*.^3^ concluded that it is easier to detect in-cell signals from disordered proteins than those originating from folded proteins and that this difference could be exploited, in principle, to distinguish between flexible parts and more rigid parts also when studying in-cell NMR of folded proteins.

Here, we have shown how we can exploit this behavior to selectively observe only specific disordered regions of proteins otherwise well ordered, and identify their flexibility. We have studied in parallel two members of the frataxin family and shown that the spectrum of the evolutionary conserved folded domain which corresponds to the full-length protein in prokaryotes is not observable in cell. Conversely, resonances from the N-terminus of the yeast ortholog which contains the mitochondrial signal can be selectively observed demonstrating that this region is unfolded and flexible. This result is interesting for various reasons. It agrees with previous studies on human frataxin which has a similar behavior despite the extensive sequence diversity of this region in the two proteins.^13^ In a solution structure (2ga5), the N-terminus consistently bends back by about 180° in all the structures of the NMR bundle and packs against the conserved globular domain.^38^ This is at variance with what we observed in the NMR spectra: the flexibility of this region is clearly supported by the relaxation parameters and by the chemical shift indices. We were also unable to identify NOEs between the N-terminal tail and helix 1 as it would be expected from the 2ga5 structure (for instance between residues Q59, E64, and E71). We can thus conclude that the N-terminus of Yfh1 is flexible and unstructured at least up to residue P62.

Another interesting outcome from our work is that our results do not support the suggestion that in-cell Yfh1 is present as an iron-induced aggregate,^21^ at least in prokaryotic cells. Even if it could be suggested that the reason why we do not observe the in-cell spectrum is the formation of such aggregates, our data do not support the presence of appreciable quantities of high molecular weight species which, if present at all, must provide only a minor contribution. The correlation time observed in the lysate is consistent with the presence of predominantly monomeric or low molecular weight species, resolving the debate on whether frataxins work as large ferritin-like aggregates.^21^ The formation of iron-induced aggregates in the cytosol is anyway unlikely because iron-promoted aggregation was mostly observed under very low ionic strengths which are very distant from the in-cell conditions.^33^ Interestingly, the residues that we detected in-cell are not visible in the crystal structure of a Yfh1 mutant (pdb id 2fql) designed to destabilize the first helix by mutating Y73 into an alanine.^23^ As a result, helix 1 in this structure is one turn shorter than what is observed in other orthologs^14^ or expected on the basis of the chemical shift indices. This region is instead expected to form a short 3_10_ helix in the wild-type.^39^

In conclusion, we have discussed an interesting application of in-cell NMR in which we have exploited to our advantage the selective observation in-cell of flexible regions of a protein to gain precious information about protein dynamics. This approach can nicely complement the more traditional relaxation data being more rapid and direct, since it does not require purification and long measurement times. Along these lines, in-cell NMR may be exploited in the future to study other interesting and challenging systems.
