# Supplementary material for: Selective observation of the disordered import signal of a globular protein by in-cell NMR: The example of frataxins
Source: Protein Sci. 2015 Apr 10;24(6):996–1003. doi: 10.1002/pro.2679 (PMC4456112; doi:10.1002/pro.2679)
Supplement: Supplementary file 1 [file pro0024-0996-sd1.doc]

**Supporting Information**

**Selective observation of the disordered import signal of a globular protein by in-cell NMR: The example of frataxins**

Annalisa Pastore, Chiara Pastore, Matija Popovic, Filippo Prischi, Domenico Sanfelice, Piero Andrea Temussi

^1^National Institute for Medical Research, MRC, The Ridgeway, London, United Kingdom

^2^Department of Clinical Neuroscience, King’s College London, Denmark Hill Campus, London, United Kingdom.

-----------------------------


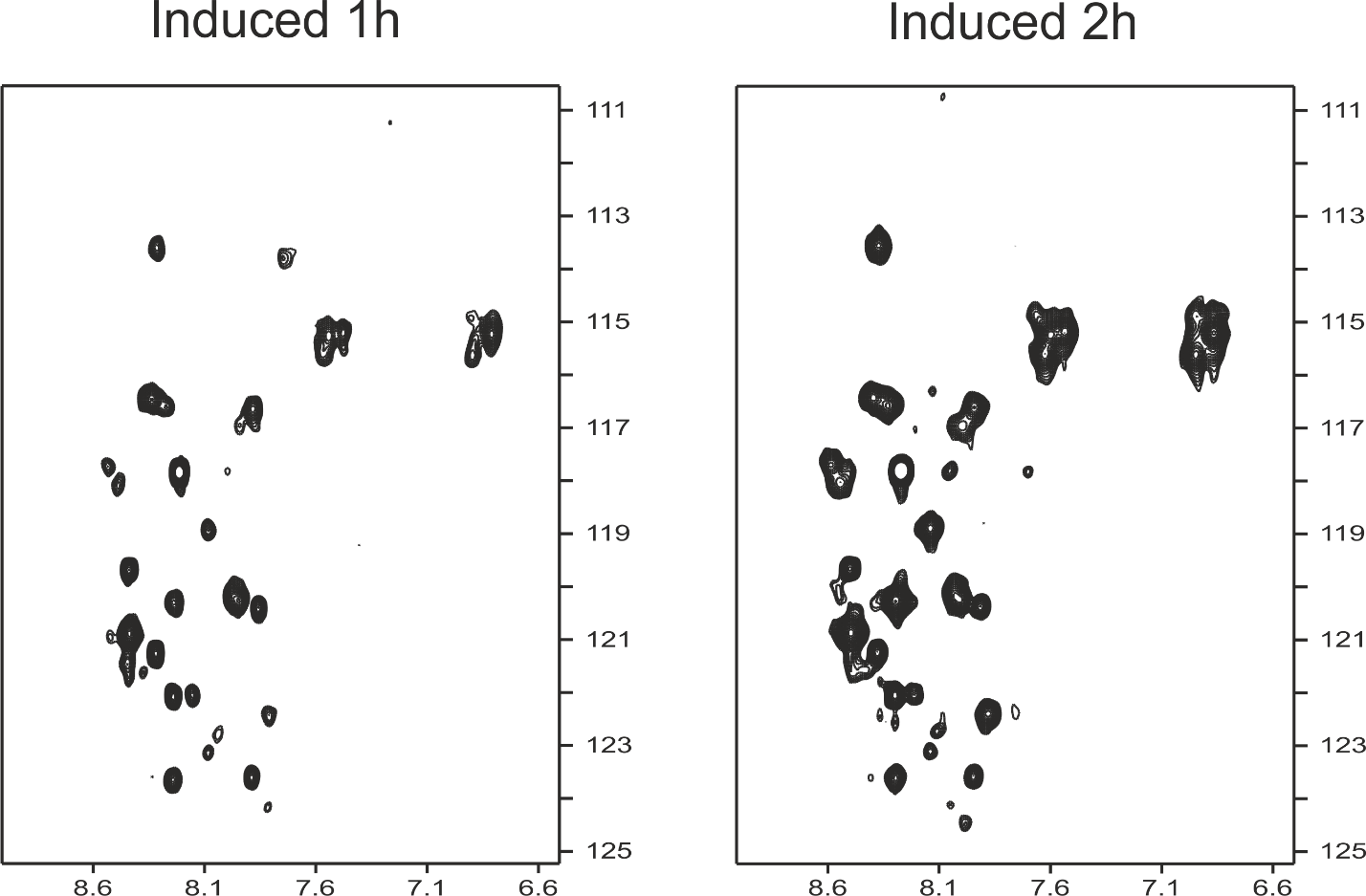


**Figure S1.** Comparison of the ^15^**N** HSQC NMR spectra of cells expressing Yfh1, after 1h (left panel) and after 2 h (right panel) of protein induction respectively.


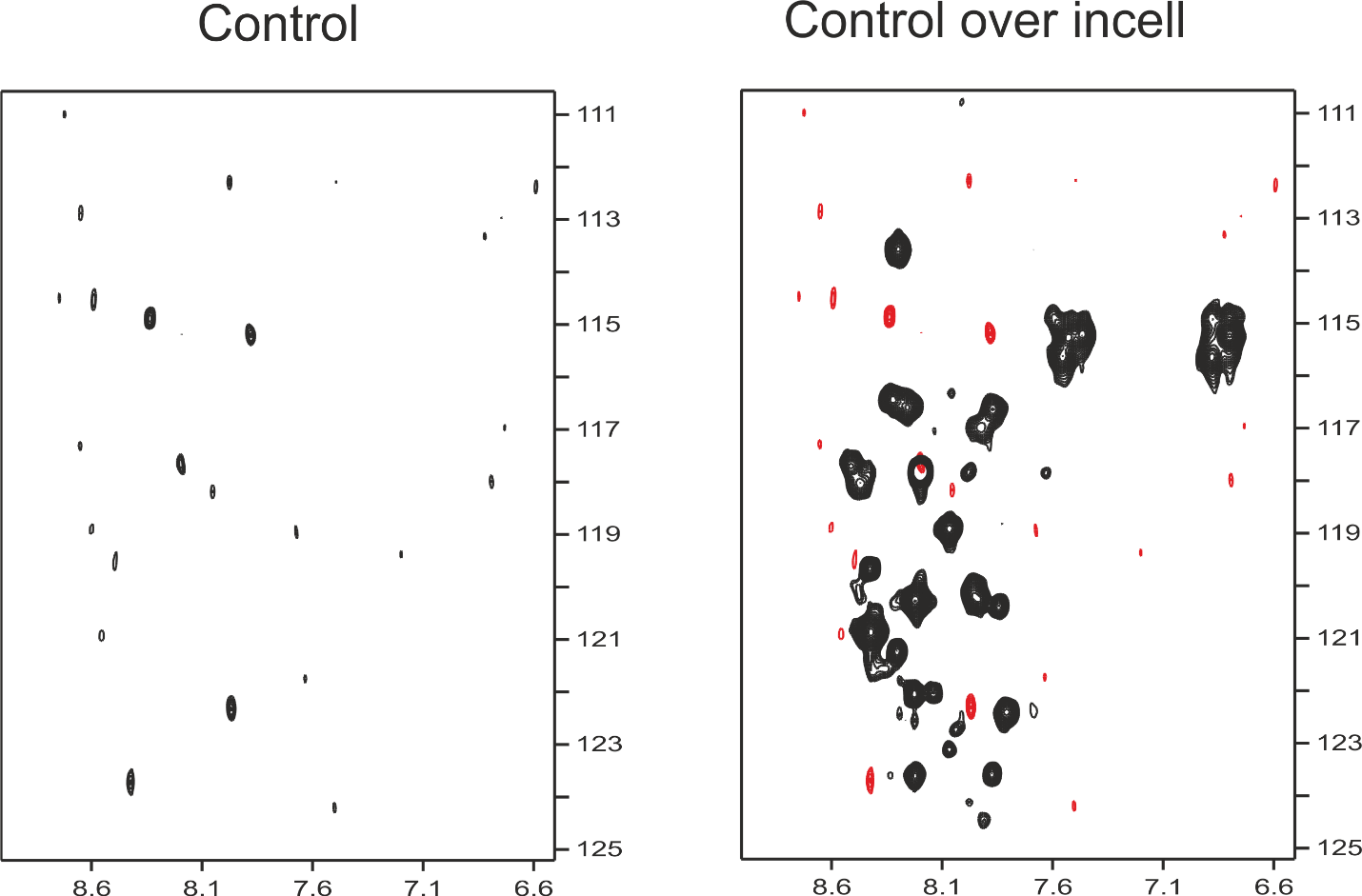


**Figure S2.** Comparison of the ^15^**N** HSQC NMR spectra of cells expressing Yfh1. (left panel): supernatant after 2h expression and the spectrum of the supernatant overlaid to the in-cell spectrum (right panel).


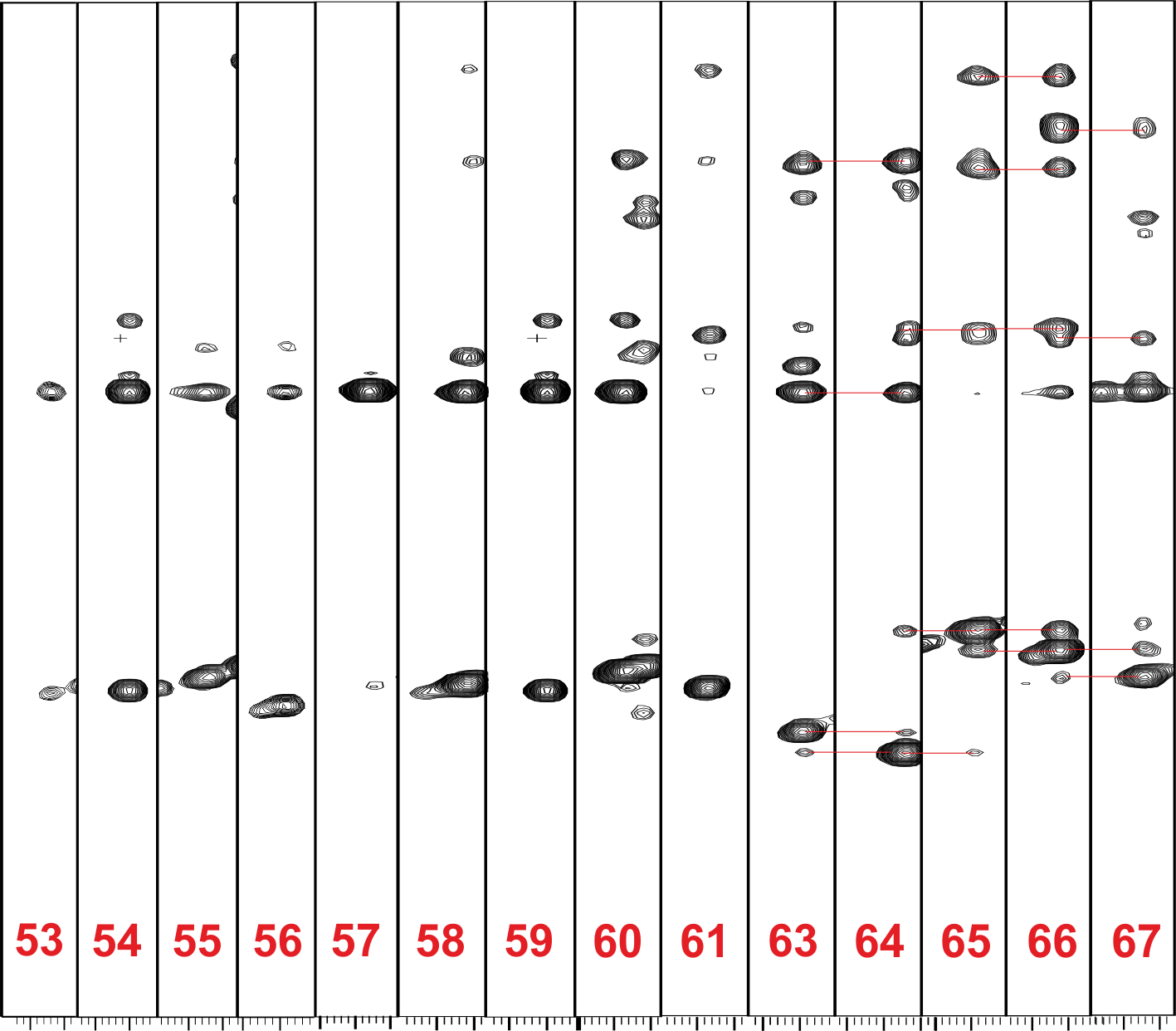


**Figure S3.** Representative strips from the 3D ^15^N NOESY NMR spectrum of Yfh1. Sequential NOEs begin at Gln 63.
